# Characterization of primary visual cortex input to specific cell types in the superior colliculus

**DOI:** 10.3389/fnana.2023.1282941

**Published:** 2023-11-10

**Authors:** Shuang Jiang, Suraj Honnuraiah, Greg J. Stuart

**Affiliations:** ^1^Eccles Institute for Neuroscience, John Curtin School of Medical Research, Australian National University, Canberra, ACT, Australia; ^2^Department of Physiology, Monash University, Clayton, VIC, Australia

**Keywords:** superior colliculus, visual cortex, vision, optogenetics, patch clamp

## Abstract

The superior colliculus is a critical brain region involved in processing visual information. It receives visual input directly from the retina, as well as via a projection from primary visual cortex. Here we determine which cell types in the superficial superior colliculus receive visual input from primary visual cortex in mice. Neurons in the superficial layers of the superior colliculus were classified into four groups – Wide-field, narrow-field, horizontal and stellate – based on their morphological and electrophysiological properties. To determine functional connections between V1 and these four different cell types we expressed Channelrhodopsin2 in primary visual cortex and then optically stimulated these axons while recording from different neurons in the superficial superior colliculus using whole-cell patch-clamp recording *in vitro*. We found that all four cell types in the superficial layers of the superior colliculus received monosynaptic (direct) input from V1. Wide-field neurons were more likely than other cell types to receive primary visual cortex input. Our results provide information on the cell specificity of the primary visual cortex to superior colliculus projection, increasing our understanding of how visual information is processed in the superior colliculus at the single cell level.

## Introduction

Visual information in mammals is processed by two main brain areas, the visual cortex and the superior colliculus (SC). Whereas the visual cortex receives visual information solely via the thalamus, the SC receives visual input directly from the retina, as well as via a projection from primary visual cortex (May, 2006). In rodents, visual information received by the SC is critical for initiating orienting movements as well as defensive behaviours (Cang et al., 2018; Basso et al., 2021; Wheatcroft et al., 2022).

Much work has been done recently to uncover the circuits underlying SC-mediated defensive behaviours in mice (Cang et al., 2018; Basso et al., 2021; Wheatcroft et al., 2022). Less attention, however, has been paid to the role of the specific cell types in the mouse SC driving these behaviours. The superficial layers of mouse SC (the stratum zonale – SZ, stratum griseum superficiale – SGS and stratum opticum – SO) are exclusively visual, receiving dense input from both the retina and the primary visual cortex (V1) (Olavarria and Van Sluyters, 1982; Harvey and Worthington, 1990; Wang and Burkhalter, 2013). While the function of the V1 to SC projection in mice is unclear, optogenetic activation of the V1 to SC projection in mouse leads to freezing behaviour (Liang et al., 2015; Zingg et al., 2017). V1-recipient SC neurons in mice send projections to the lateral posterior nucleus of thalamus (LP), with optogenetic activation of the axon terminals of these neurons triggering freezing behaviour (Zingg et al., 2017). These data suggest that the V1-SC-LP pathway in mice is important for driving freezing in response to visual threats. As SC projections to LP in mice arise solely for wide-field neurons (Gale and Murphy, 2014), this data suggests an important role of wide-field neurons in driving freezing behaviour via the V1-SC-LP pathway. Consistent with this idea, a recent study has suggested that V1 projections primarily target wide-field neurons, and not GABAergic inhibitory neurons in the superficial SC (Masterson et al., 2019), however, the specific cell types in the SC that receive direct input from V1 is currently unknown.

The current study builds on earlier work showing that there are four main cell types with distinct morphological and electrophysiological properties in the superficial layers of mouse SC. Specifically, these are wide-field, narrow-field, stellate and horizontal neurons (Langer and Lund, 1974; Gale and Murphy, 2014). To determine which of these four main cell types receive input from V1, we optogenetically activated V1 terminals in brain slices of mouse SC while recording neuronal activity in SC neurons using whole-cell patch clamp recording *in vitro*. To visualize dendritic morphology SC neurons were filled with biocytin and their dendritic trees reconstructed *post hoc* based on confocal fluorescence images. Using methods similar to the earlier work by Gale and Murphy (2014), we classified SC neurons into four groups based on their electrophysiological and morphological properties. We find that all four cell types in the superficial SC received direct (monosynaptic) input from V1, with wide-field neurons receiving more powerful V1 input than any other cell type.

## Materials and methods

### Mice

All protocols involving animals were approved by the Australian National University Animal Experimentation Ethics Committee. The mouse line used in this study was C57BL6/J (from the Australian Phenomics Facility at the Australian National University).

### Viral injections

Mice at 4 weeks age were anesthetized with isoflurane (3% in oxygen) in an induction chamber and then transferred to a stereotaxic frame where anaesthesia was continued via a close-fitting nosepiece (1% isoflurane in oxygen). Anaesthesia was confirmed by the absence of a response to a foot pinch. Ear bars together with a nose clamp were used to stabilize the head. A rectal probe coupled to a feedback-controlled heating pad was used to maintain body temperature at 37°C. The scalp was shaved and the remain hair removed with hair-removal cream. After cleaning with antiseptic, an incision was made along the midline of the scalp to expose the skull and a small craniotomy (0.5 mm diameter) made using a dental drill over V1 in the left hemisphere (V1 coordinates based on the Allen Mouse Brain Atlas: 0.5 mm anterior to bregma; 2.5 mm lateral from midline).

Viral injections were made using a glass pipette (tip diameter ~ 20 μm) back-filled with silicone oil and tip-filled with 2 μL of adeno-associated virus (AAV) containing ChR2 (AAV1-hSyn.ChR2(H134R)-eYFP.hGH; AddGene). A total volume 350 ~ 450 nL was injected into V1 at a depth of 350 μm to 750 μm in 23 nL increments, with a two-minute delay between injections. After viral injection, the pipette was slowly removed and the scalp sutured. To prevent infection, antiseptic was applied to the wounded area. Mice were injected subcutaneously with 0.2 mL Meloxicam (0.1 mg/mL) for pain management, recovered in a warm cage with wet food and water and were monitored for 3 days (twice per day) to make sure that they fully recovered from surgery.

### Brain slice recordings

Brain slices of the left hemisphere containing V1 and SC were prepared 3 to 4 weeks after viral injection of ChR2. Mice (age 7 to 8 weeks) were deeply anesthetized with isoflurane (3% in oxygen) and immediately decapitated. The brain was rapidly extracted and immersed in an ice-cold cutting solution of composition: 100 mM Choline Chloride, 11.60 mM N-ascorbate, 7 mM MgCl_2_, 3.10 mM Na-pyruvate, 2.50 mM NaH_2_PO_4_, 0.50 mM CaCl_2_ (pH = 7.4). Brain slices (300 μm thick) containing the left V1 and SC were cut in a coronal plane using a Leica Vibratome 1000S. Slices were transferred to warm (35°C) incubating solution of composition: (92 mM NaCl, 2.5 mM KCl, 1.2 mM NaH_2_PO_4_, 30 mM NaHCO_3_, 20 mM HEPES, 25 mM glucose, 3 mM sodium pyruvate, 2 mM MgSO_4_, 2 mM CaCl_2_) for 30 min, which was then allowed to gradually cool to room temperature for another 30 min before being used for recording.

During recording slices were continuously perfused with oxygenated, heated (34°C) artificial cerebral spinal fluid (ACSF) of composition: 125 mM NaCl, 25 mM NaHCO_3_, 3 mM KCl, 1.25 NaH_2_PO_4_, 2 mM CaCl_2_, 1 mM MgCl_2_ and 25 mM glucose (pH = 7.4; bubbled with 95% O_2_ / 5% CO_2_). The region of SC containing V1 projections was identified by the presence of green fluorescent. Whole-cell somatic recordings were performed under visual control using a 60x objective on an Olympus BX50 microscopy equipped with differential interference contract optics and infrared illumination (Stuart et al., 1993). Patch pipettes with an open tip impedance of 5 to 7 MΩ were filled with an internal solution containing: 10 mM KCl, 130 mM K-Gluconate, 10 mM HEPES, 4 mM Mg^2+^ATP, 0.3 mM Na_2_GTP, 10 mM Na_2_Phosphocreatine and 0.5% biocytin (pH = 7.25; KOH). All recordings were made in current-clamp mode using a current clamp amplifier (Dagan BVC-700A). To characterize electrophysiological properties of SC neurons, hyperpolarizing and depolarizing current steps of 1,000 ms duration (from −400 pA to +600 pA, 50 pA/step) were applied via the somatic recording pipette. Voltage signals were filtered at 10 kHz and digitized at 20 or 50 kHz using an A-D converter (ITC-18, Instrutech/HEKA, Germany) under the control of Axograph acquisition software (Axograph X, Axograph Scientific, Sydney, Australia) running on an Apple Macintosh computer (iMAC-i7). Axograph was used for both data acquisition and primary analysis.

Full-field optogenetic stimulation was performed with 470 nm blue light pulses (2 ms duration) via an LED light source (ThorLab, United States) attached to the epifluorescence port of the microscope. A GFP (green fluorescent protein) filter cube (Chroma) was inserted into the light path to direct 470 nm LED light onto the brain slice. For each neuron we averaged the response to 10 trails of LED stimulation at different intensities (0.7 mW, 1.4 mW, 1.9 mW, 2.5 mW, 2.9 mW and 4.8 mW).

In some experiments, one or more the following pharmacological agents was added to the ACSF perfusing the slice: The sodium-channel blocker tetrodotoxin (TTX; 1 μM; Hello Bio), the potassium-channel blocker 4-aminopyridine (4-AP, 100 μM, Sigma), the α-amino-3-hydroxy-5-methyl-4-isoxazolepropionic acid (AMPA) receptor blocker 6,7-dinitroquinoxaline-2,3-dione (DNQX; 10 μM; Tocris) and the N-methyl-D-aspartate (NMDA) receptor blocker amino-5-phosphonovaleric acid (APV; 25 μM; Tocris).

### Immunohistochemistry

Fluorescence labelling was used to obtain the morphology of recorded neurons (Suzuki and Bekkers, 2010). Recorded neurons were filled with biocytin (0.5%) during electrophysiological recording. A minimum recording time of 10 min was required to allow adequate biocytin-filling. At the end of recording, the recording pipette was removed slowly in small steps to keep the soma intact. Slices were then fixed in 4% paraformaldehyde, covered with aluminium foil and placed in the refrigerator (4°C) overnight. Fixed slices were washed in PBS (phosphate buffered saline, pH = 7.4; three washes at 10 min intervals) and treated with PBS plus 0.3% Triton X-100 and 1% BSA (bovine serum albumin) for 1 h. Slices were then incubated for 18 h (16–24 h) with Streptavidin-Alexa Fluor 594 (dilution 1:1,000) in the refrigerator (4°C). Following further washes in PBS (three washes at 10 min intervals), slices were mounted on glass slides using fluorescence mounting medium. A confocal microscope (Zeiss LSM 800 with Airyscan) was used for visualization of biocytin-filled neurons. Neurolucida 10 was used for morphological tracing.

### Analysis of morphological properties

Scholl analysis and polar plots of dendritic length were performed on reconstructed neurons. Total dendritic length in the upper and lower vertical quadrants (*VQ1, VQ2*) and left and right horizontal quadrants (*HQ1, HQ2*) was calculated, where *VQ1* = polar angle 45 to 135 degrees, *VQ2* = polar angle 225 to 315 degrees, *HQ1* = polar angle 135 to 225 degrees, *HQ2* = polar angle 315 to 45 degrees relative to the pia (polar angle 0 or 180 degrees). Dendritic orientation index (*DOI*) was calculated by subtracting the total dendritic length in vertical (*VQ1 + VQ2*) from horizontal (*HQ1 + HQ2*) quadrants, divided by the total dendritic length in all quadrants.

Morphological classification proceeded as follows. First, neurons were classified as horizontal based on a negative *DOI*. Out of the remaining neurons, wide-field neurons were classified by subtracting the total dendritic length in the upper vertical quadrant (*VQ1*) from the lower quadrant (*VQ2*) and applying a threshold. All the neurons above the threshold were classified as wide-field neurons. We then calculate the *DIO* of the remaining neurons, with neurons with a *DOI* above a threshold classified as narrow-field and those below classified as stellate neurons (see Results).

### Analysis of electrophysiological properties

We determined the following electrophysiological properties. Resting membrane potential (parameter 1) and input resistance (parameter 2) measured at steady state during long (1,000 ms) hyperpolarizing current pulses. Sag in the membrane voltage back to the resting membrane potential during long (1,000 ms) hyperpolarizing current pulses (parameter 3). The sag ratio was defined as the steady-state voltage at the end of hyperpolarizing current pulses divided by the peak voltage during hyperpolarizing current injections (−200 pA). The sag width, defined as time elapsed from the peak hyperpolarization during hyperpolarizing current injections (−200 pA) and development of the sag to half of its maximum amplitude (parameter 4). The amplitude of the rebound depolarization following cessation of long hyperpolarizing current injections (1,000 ms, −200 pA) (parameter 5), defined as the rebound potential. The maximum number of rebound spikes evoked at the cessation of long hyperpolarizing current injections (1,000 ms, −200 pA) (parameter 6). Action potential (AP) properties were measured from the first AP evoked by the lowest current injection which caused the neuron to fire APs regularly. AP threshold was defined as the membrane potential at which the slope of the AP reached 50 mV/ms (parameter 7). The voltage reached at the peak of the AP (AP height) measured from AP threshold (parameter 8). AP half-width, defined as the width of the AP at half AP height (parameter 9). The AP afterhyperpolarization measured as the voltage difference between AP threshold and the maximum hyperpolarizing potential after an AP (parameter 10). The maximum firing rate for the largest, positive current injection was also determined (parameter 11).

### Hierarchical cluster analysis

Hierarchical clustering, also known as hierarchical cluster analysis, was performed in MATLAB using the 11 electrophysiological parameters described above, as well as the distance of the soma of the recorded neuron from the SC surface (parameter 12). Prior to hierarchical clustering, each parameter was normalized to give a range between 0 and 1. Hierarchical clustering starts by treating each cell as a separate cluster. It then repeatedly identifies clusters with similar properties and merges them. This continues until all the clusters are merged. The output of hierarchical clustering is a dendrogram, which shows the Ward’s linkage distance between clusters. Ward’s linkage distance is calculated from the average squared Euclidian distance between cluster centres.

### Statistics

To determine whether a recorded SC neuron responded to optogenetic stimulation or not, we used non-parametric, receiver operating characteristic (ROC) analysis. SC neurons that showed a response to optogenetic stimulation were considered as “responders”, whereas those that did not show a response were considered as “non-responders”. ROC analysis estimates whether an ideal observer could classify whether a response was recorded in one of two possible conditions (here optogenetic stimulation or not). In each cell we compared the amplitude of the response 20 ms after optogenetic stimulation onset (“light”) with that observed 20 ms prior to optogenetic stimulation (“baseline”). Trials were filtered using a running (100 point) average prior to measurement of “baseline” and “light” response amplitude to remove noise. The overlap between “baseline” and “light” distributions was quantified by applying criterion levels ranging from the minimum to the maximum. Plots of the fraction of “baseline” and “light” responses above a given criterion level were used to generate ROC curves for each cell. The area under the ROC curve (AUC) represents a measure of separability between the “baseline” and “light” distributions. Statistical significance of the obtained AUC curves was determined by bootstrap analysis. 1,000 synthetic ROC curves were calculated based on values drawn randomly drawn from the “baseline” and “light” data set for each cell. The fraction of synthetic ROC curves with AUC values greater than that experimentally observed indicates the level of significance. If the observed AUC value was greater than 95% of the synthetic AUC values, we classified the cell as a “responder”. Conversely, if the observed AUC value was less than 95% of the synthetic AUC values, the neuron was classified as a “non-responder”.

For statistical comparison of properties between different cell types, an ANOVA was first used to determine whether there was an overall difference. If a difference was observed, a pairwise multiple comparison post-hoc test using Tukey’s method was used to determine which groups were different. Statistical variance for the post-hoc test was estimated from the variability across all groups. Statistical significance was set at *p* < 0.05. Results were presented as mean values ± the standard error of the mean (SEM), unless otherwise stated. In the figures, a statistical difference of *p* < 0.05 is indicated by one asterisk; *p* < 0.01 by two asterisks and *p* < 0.001 by three asterisks. Non-significant difference is indicated by “ns”.

## Results

### Effective ChR2 expression and optogenetic activation in V1 and SC

**Figure 1:**
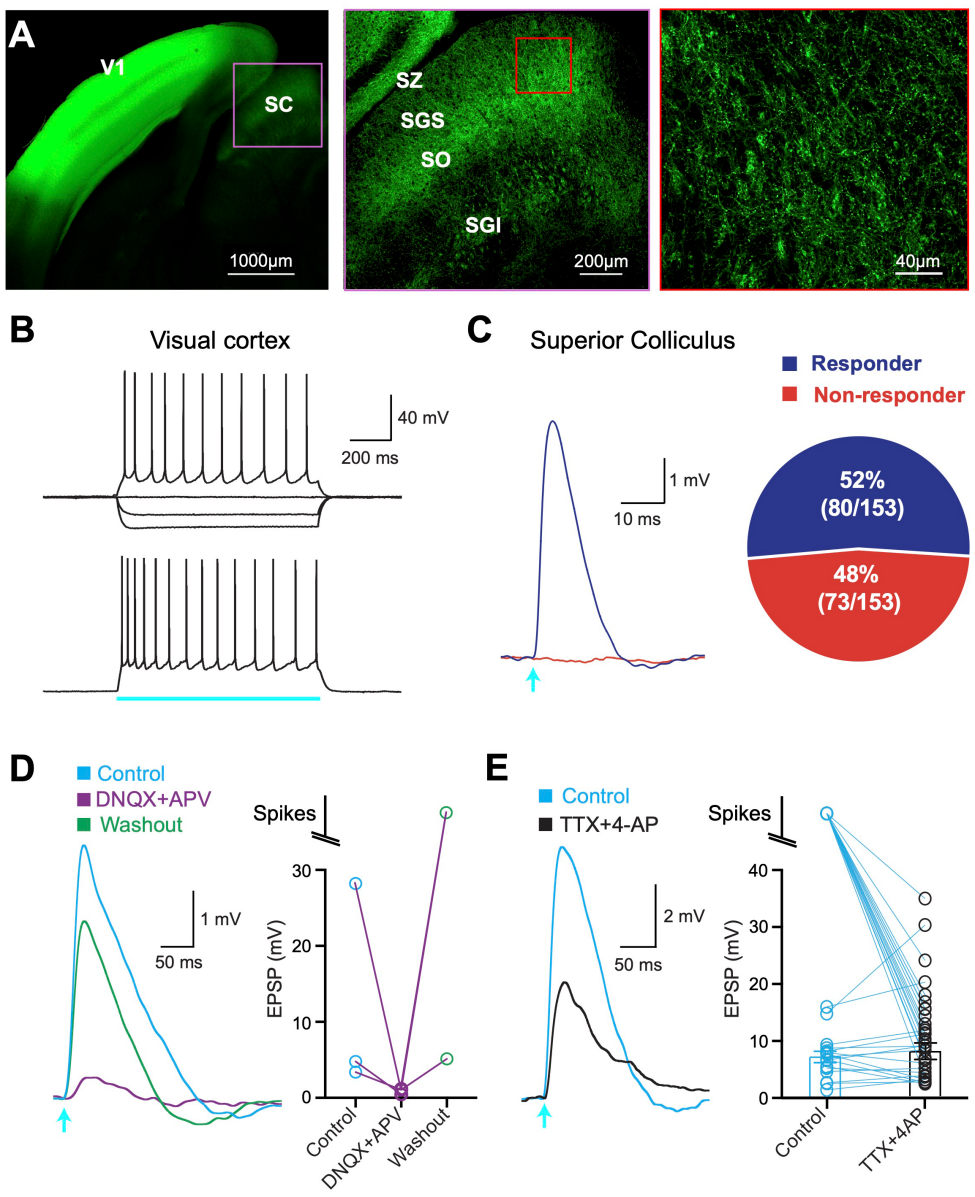
ChR2 expression and optogenetic activation. **(A)** Left: Confocal image of a brain slice showing ChR2-eYFP expression in superior colliculus (SC) following viral injection into primary visual cortex (V1). Middle: Higher magnification of the region delineated by the pink box in the left image showing ChR2-eYFP expression in all major divisions of the SC. Right: Higher magnification of the region delineated by the red box in the middle image. SZ, stratum zonale; SGS, stratum griseum superficiale; SO, stratum opticum; SGI, stratum griseum intermedium. **(B)** Example response of a cortical pyramidal neuron in V1 to somatic current injection (top; −200pA, −100 pA and + 200pA) and optogenetic stimulation (bottom; 1,000 ms, 1.4 mW; blue bar). **(C)** Left: Neuron in the superficial SC responding to brief (2 ms) optogenetic stimulation (arrow; 2.9 mW; “responder”) and one that did not (red; “non-responder”). Right: Pie chart showing the percentage of all 153 recorded neurons in the superficial SC that responded to optogenetic stimulation (purple) and those that did not (red). **(D)** Left: Response in a superficial SC neuron to brief (2 m) optogenetic stimulation (arrow; 2.9 mW) in control (blue), DNQX+APV (purple) and after washout (green). Right: Summary data showing the impact of DNQX+APV. **(E)** Left: Response in a superficial SC neuron to brief (2 ms) optogenetic stimulation (arrow; 2.9 mW) in control (blue) and the presence of TTX + 4-AP (black). Right: Summary of response amplitude (or spiking) in superficial SC neurons in control (blue) and TTX + 4-AP (black). Circles represent individual neurons; bars represent the mean (excluding spikes).

To characterize the V1 projection to the SC fluorescently-tagged ChR2 (AAV1-hSyn.ChR2(H134R)-eYFP.hGH) was injected into V1 in one hemisphere of 4-week-old mice. After waiting 3 to 4 weeks for viral expression, a band of fluorescence could be visualized in V1 with less intense fluorescence in the SC (Figure 1A, left). Closer examination indicated that V1 projections to the SC were found predominantly in the superficial and intermediate layers of SC (Figure 1A, middle). Images at higher magnification indicated clear expression of fluorescence in axons and boutons in superficial SC (Figure 1A, right).

To determine whether neurons in V1 expressed ChR2 we made whole-cell recordings from the somata of pyramidal neurons in V1 (Figure 1B). Action potential firing was observed in response to optogenetic stimulation of the soma and dendrites of pyramidal neurons in V1 (duration: 1,000 ms; intensity: 1.36 mW), confirming functional expression of ChR2 (Figure 1B). Recordings were then made from neurons in the superficial layers of the SC (SGS and SO; up to ~500 μm from the SC surface) to investigate whether SC neurons received input from V1. Brief (2 ms) optogenetic activation of V1 axons in SC evoked subthreshold responses or action potentials in 52% of SC neurons located in the SGS and SO (Figure 1C; 80 out of 153 cells), whereas responses were not observed in 48% of SC neurons (73 out of 153) even when tested at the highest LED power available (Figure 1C; LED intensity: 4.8 mW). We classified cells that elicited a response during brief (2 ms) optogenetic activation as “responders,” whereas those that did not were classified as “non-responders” (see Methods).

To confirm that responses in SC neurons were synaptic rather than due to ChR2 self-expression we bath applied the AMPA and NMDA glutamate antagonist DNQX and APV. In all cases, optogenetic responses were essentially abolished in the presence of DNQX and APV and recovered after washing out (Figure 1D). These experiments confirm that optogenetic responses in SC neurons are synaptic in origin and due to the release of glutamate activating AMPA and NMDA receptors on SC neurons.

Finally, we tested if synaptic responses were monosynaptic. To do this we bath applied the sodium channel blocker TTX together with the potassium channel blocker 4-AP (Petreanu et al., 2009). TTX will block action potential initiation, thereby blocking synaptic responses due to activation of surrounding neurons. By blocking potassium channels in axons, 4-AP enhances transmitter release from ChR2-expressing V1 terminal. Under these recording conditions synaptic responses can only be evoked by expressing ChR2 V1 terminals that synapse directly onto SC neurons. All polysynaptic input to SC neurons will be blocked. In all cases, light-evoked responses remained in the presence of TTX and 4-AP (Figure 1E; *n* = 39). This result indicates that all SC neurons that responded to optogenetic activation under control conditions received direct, monosynaptic input from V1.

### Four cell types in the SC with distinct morphological and electrophysiological properties

**Figure 2:**
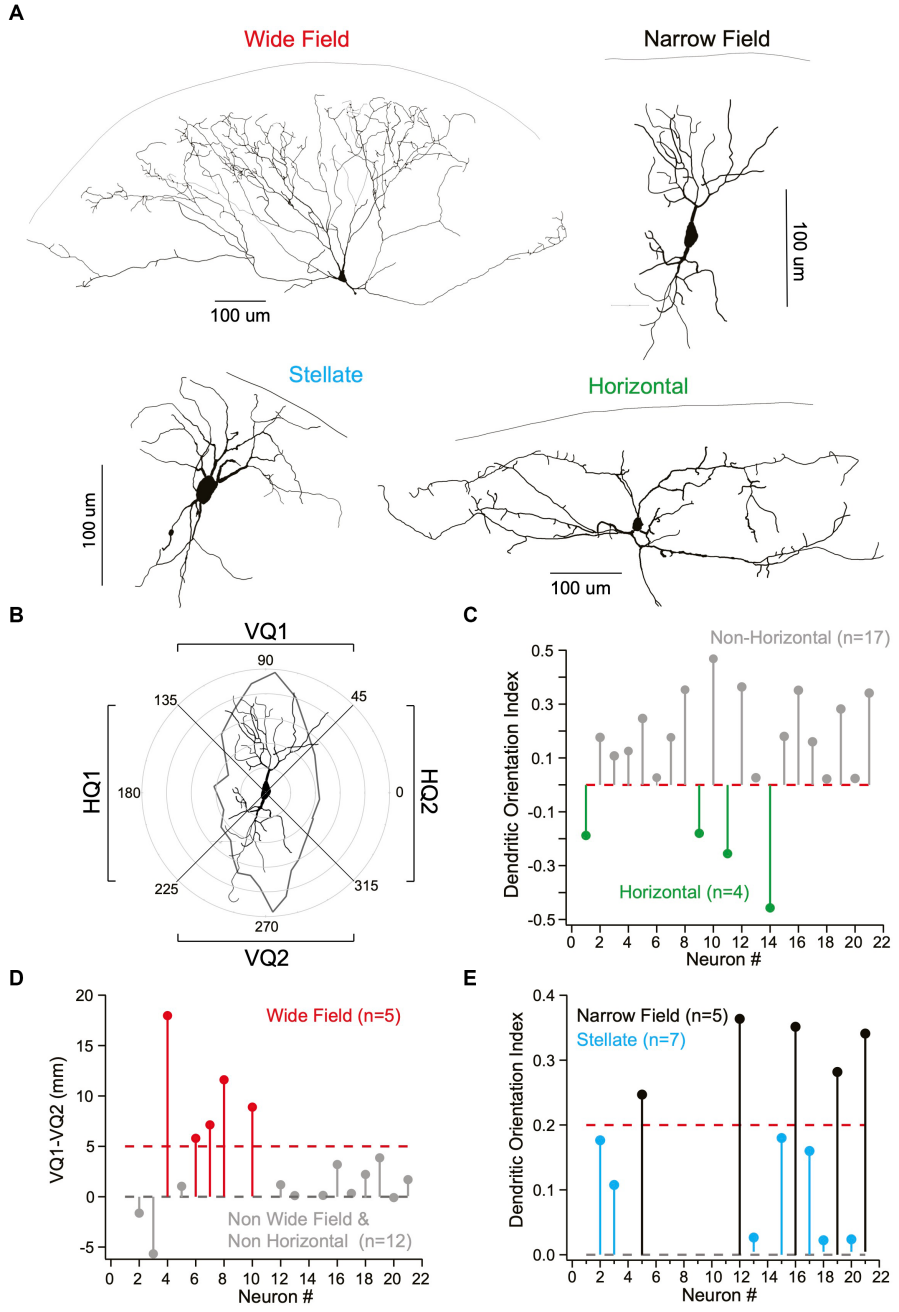
Identification of different cell types in superficial superior colliculus based on their morphology. **(A)** Representative examples of the four main neuron cell classes in superficial superior colliculus based on Neurolucida reconstructions. Top, left: Wide-field neuron (widespread “fan-like”/conical dendrites). Top, right: Narrow-field neuron (vertically spanning dendrites perpendicular to pia). Bottom, left: Stellate neuron (uniform, star-like/spherical dendritic architecture). Bottom, right: Horizontal neurons (lateral spanning dendrites parallel to pia). **(B)** Classification on neurons into different classes was based on quantification of polar plots using the total dendritic length in the upper and lower vertical quadrants (VQ1 and VQ2, respectively) and left and right horizontal quadrants (HQ1 and HQ2, respectively). A narrow-field neuron with dendrites primarily in the upper and lower vertical quadrants (VQ1 and VQ2) is shown as an example. **(C)** Dendritic orientation index (DOI) was calculated by subtracting total dendritic length in vertical (VQ1 + VQ2) from horizontal quadrants (HQ1 + HQ2) then dividing by the total dendritic length in all quadrants. Cells were classified as horizontal neurons (green; *n* = 4) if the DOI was negative. **(D)** Wide Field neurons (red; *n* = 5) were identified by subtracting the total dendritic length in the upper vertical quadrant (VQ1) from the lower vertical quadrant (VQ2) then applying a threshold (dotted red line). **(E)** The DOI of the remaining neurons was used to differentiate narrow-field (black; *n* = 5) from stellate neurons (cyan; *n* = 7) based on an arbitrary threshold (dotted black line) under the assumption that stellate neurons with a spherical dendritic tree will have an DOI close to zero.

As the four main cells types in the superficial SC have previously been characterized based on their dendritic morphology, we reconstructed the dendritic morphology of a subset of recorded neurons following filling with biocytin and fluorescence labelling (Figure 2A; *n* = 21). To characterize their dendritic morphology, we determined the total dendritic length in the upper and lower vertical and right and left horizontal quadrants for each cell (Figure 2B; see Methods). This information was used to determine the dendritic orientation index (DOI) of each cell (Gale and Murphy, 2014), where the DOI is the total dendritic length in the vertical minus the horizontal quadrants, divided by the total dendritic length in all quadrants (see Methods). Cells with a negative DOI, and therefore dendrites primarily in the horizontal quadrants, were classified as horizontal neurons (Figure 2C; *n* = 4). For the remaining neurons (*n* = 17) we subtracted the total dendritic length in the lower vertical quadrant from that in the upper vertical quadrant. If the total dendritic length in the upper vertical quadrant was five times greater or more than that in the lower vertical quadrant we classified neurons as wide-field (Figure 2C; *n* = 5). We then re-examined the DOI of the remaining neurons (*n* = 12) and again applied a threshold (in this case 0.2), with all neurons with a DOI less than this value classified as stellate neurons (Figure 2D; *n* = 7). The rational here is that neurons with a circular/spherical dendritic tree, such as stellate neurons, would be expected to have similar dendritic lengths in all four quadrants and therefore a DOI close to zero. By default, the remaining neurons, which had a DOI above 0.2, were classified as narrow-field neurons (Figure 2E; *n* = 5).

**Figure 3:**
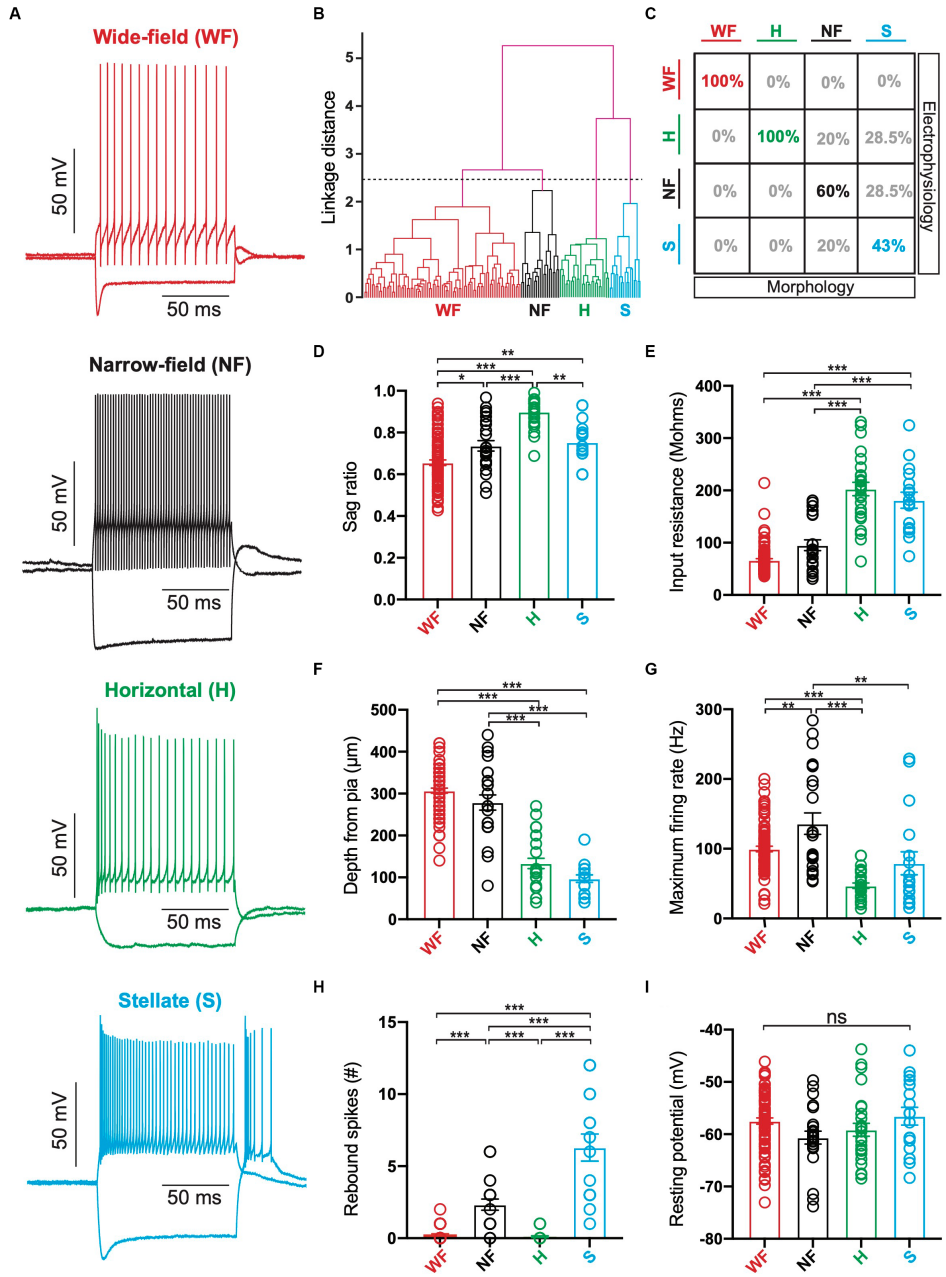
Identification of different cell types in superficial superior colliculus based on their electrophysiological properties. **(A)** Responses to hyperpolarizing (−200 pA) and depolarizing (+100 pA) somatic current injections in neurons from each of the four classed. Red: Wide-field (WF) neurons; Back: Narrow-field (NF) neurons; Green: Horizontal (H) neurons; Cyan: Stellate (S) neurons. **(B)** Dendrogram of hierarchical clustering of electrophysiological properties. Black dashed line indicates the linkage distance for 4 clusters (WF neurons: *n* = 85; NF neurons: *n* = 24; Horizontal neurons: *n* = 27; Stellate neurons: *n* = 17). **(C)** Table of the percentage of superficial SC neurons defined by morphological properties (columns) that were classified as the same or a different cell type based on hierarchical clustering of electrophysiological properties (rows). **(D–I)**: Histograms of the sag ratio **(D)**, input resistance **(E)**, depth of somata from pia **(F)**, maximum firing rate **(G)**, maximum number of rebound spikes after hyperpolarizing current injection **(H)** and resting membrane potential **(I)** for neurons classified as WF, NF, Horizontal or Stellate based on hierarchical clustering of electrophysiological properties (as well as soma depth). Circles represent individual neurons; bars represent the mean; error bars represent SEM. * *p* < 0.05; ** *p* < 0.01; *** *p* < 0.001; ns, not significant (Tukey post-hoc test).

In addition to this morphological analysis, we used unsupervised hierarchical cluster analysis to classify superficial SC neurons into different cell types based their electrophysiological properties as well as the soma depth from the pia (*n* = 153). In total, twelve different parameters were used for this cluster analysis (see Methods). Based on the earlier work by Gale and Murphy (2014), we classified neurons into four groups using a linkage distance of 2.5 (Figures 3A,B). As a result, we classified 85 neurons as putative wide-field, 24 as putative narrow-field, 27 as putative horizontal and 17 as putative stellate neurons. Comparison of this cluster analysis with our morphological analysis indicated that the probability that putative wide-field (*n* = 5) and horizontal neurons (*n* = 4) were grouped into the same class based on morphological and electrophysiological properties (as well as soma depth) was 100% (Figure 3C). The matching rate was lower for narrow-field and stellate neurons, however, with only 60% of narrow-field neurons (3 out of 5) and 43% of stellate neurons (3 out of 7) as defined by their morphological properties classified into the same group following cluster analysis (Figure 3C). These data suggest that the hierarchical cluster analysis does a good job of characterising wide-field and horizontal cells, whereas our capacity to differentiate between narrow-field and stellate neurons would appear to be less robust. Why this is the case is not clear, but could be due to similarities in the morphological or the electrophysiological properties of these two cell types (or both).

We next compared six of the parameters used in the hierarchical cluster analysis across the different clusters. Specifically, we tested for differences in the sag ratio, input resistance, maximum firing rate, number of rebound spikes, soma depth from the pia and the resting membrane potential between clusters (Figures 3D–I). This analysis indicated that putative wide-field neurons (Figure 3A; *n* = 85) had the largest sag ratio in response to hyperpolarizing current injections (Figure 3D; 65 ± 0.01), lowest input resistance (Figure 3E; 67 ± 3 MΩ) and were on average located at the greatest distance from the pia primarily within the lower SGS and upper SO (Figure 3F; 307 ± 6 μm). These findings are consistent with earlier work (Endo et al., 2008; Gale and Murphy, 2014). Putative narrow-field neurons (Figure 3A; *n* = 24) also had somata located primarily in deeper regions of the SGS (Figure 3F; 279 ± 18 μm) and had the highest maximum firing rates (Figure 3G; 136 ± 15 Hz), also consistent with earlier work (Gale and Murphy, 2014). Similar to wide-field neurons, putative narrow-field neurons had a relatively low input resistance (Figure 3E; 95 ± 10 MΩ), but could exhibit rebound spikes following cessation of hyperpolarizing current injections (Figure 3H). Putative horizontal neurons (Figure 3A; *n* = 27) had somata located primarily in the upper SGS (Figure 3F; 134 ± 12 μm), had the smallest sag ratio (Figure 3D; 0.90 ± 0.01) and highest input resistance (Figure 3E; 203 ± 13 MΩ). They also had the lowest maximum firing rates (Figure 3G; 47 ± 4 Hz) and were the only cell type that could not sustain firing rates above ~100 spikes/s (Figure 3G), again consistent with earlier work (Gale and Murphy, 2014). Putative stellate neurons (Figure 3A; *n* = 17) were also located more superficially in the upper SGS (Figure 3F; 97 ± 9 μm). They had relatively high input resistance (Figure 3E; 181 ± 15 MΩ) and low maximum firing rates (Figure 3G; 79 ± 16 Hz). Putative stellate neurons also exhibited the highest number of rebound spikes following cessation of hyperpolarizing current injection (Figure 3H; 6 ± 0.9). In contrast to earlier work (Gale and Murphy, 2014), no statistically significant differences in resting membrane potential was observed between the four cell types (Figure 3I). Together, these findings indicate fundamental differences between the morphology and electrophysiology of the four main cell types in the superficial SC.

### All cell types in the SC receive direct V1 input

**Figure 4:**
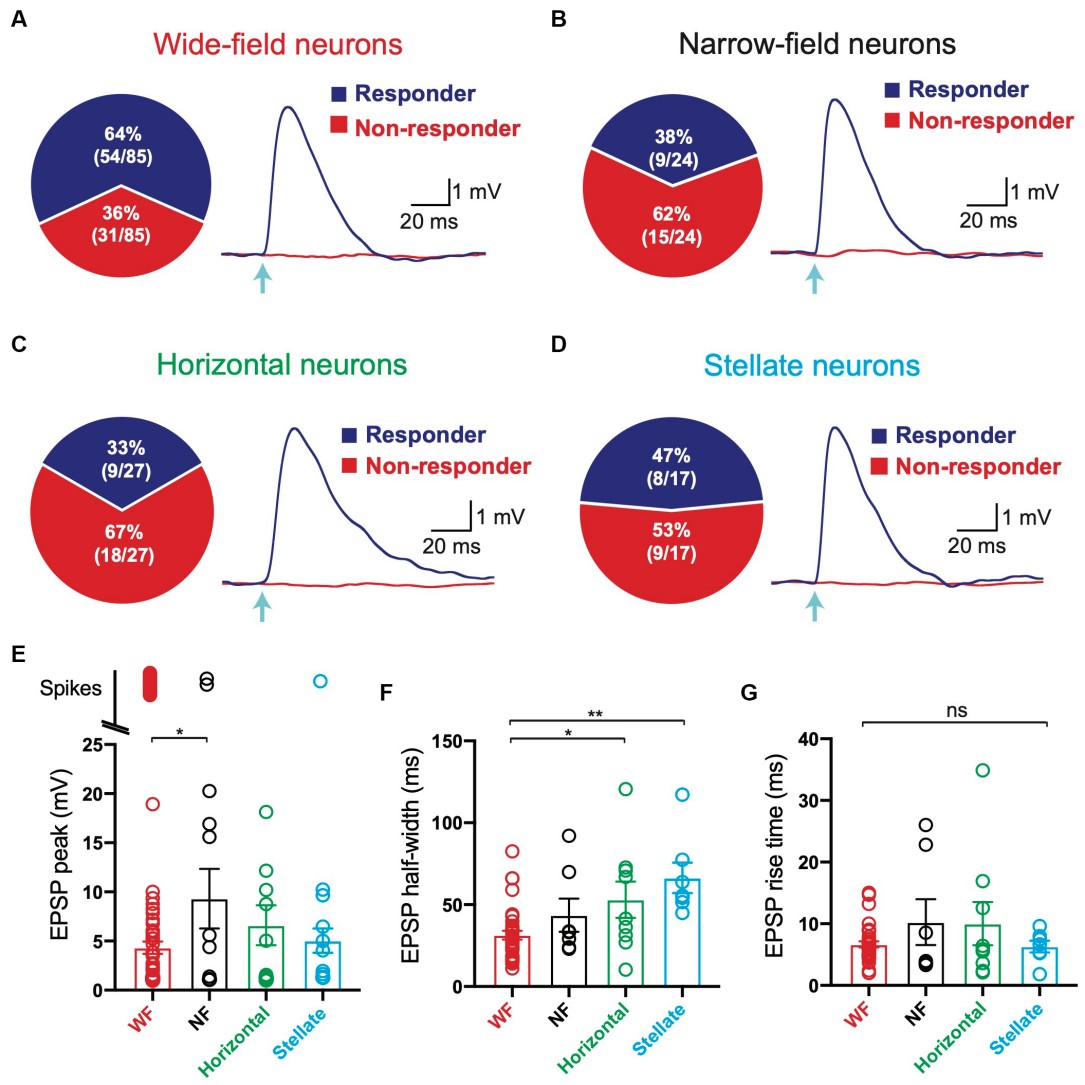
All four main cell types in the superficial superior colliculus based on hierarchical clustering of electrophysiological properties receive input from V1. **(A–D)** Left: Pie charts of the percentage of Wide Field **(A)**, Narrow Field **(B)**, Horizontal **(C)** and Stellate **(D)** neurons (based on hierarchical clustering of electrophysiological properties) receiving input from V1 (blue) compared to those that did not (red). Right: EPSPs in Wide Field **(A)**, Narrow Field **(B)**, Horizontal **(C)** and Stellate **(D)** neurons receiving input from V1 (“Responder”) and cells with no response (“non-responder”) during brief (2 ms) optogenetic activation (arrow; 2.9 mW) under control conditions. **(E,F)** Summary histograms of EPSP amplitude excluding spikes **(E)**, EPSP half-width **(F)** and EPSP rise time **(G)** in the four main cell types in superficial superior colliculus (based on hierarchical clustering of electrophysiological properties) in response to optogenetic activation of V1 axon terminals under control conditions (2 ms; 2.9 mW). Circles represent individual neurons; bars represent the mean; error bars represent SEM. * *p* < 0.05; ** *p* < 0.01; ns, not significant (Tukey post-hoc test).

We next investigated which of the four main cell types in the SC receiving V1 input. Presynaptic V1 axons were optogenetically activated while recording from different SC neurons, as described in Figure 1. In these experiments, we found that V1 axons terminated onto all four cell types rather than targeted a specific cell type in the superficial SC. On average, 64% of wide-field neurons (54 out of 85) responded to optogenetic stimulation of V1 terminals (Figure 4A), whereas 38% of narrow-field (Figure 4B; 9 out of 24), 33% of horizontal (Figure 4C; 9 out of 27) and 47% of stellate neurons (Figure 4D; 8 out of 17) responded to optogenetic stimulation of V1 terminals. As indicated earlier, when tested all responses were maintained in the presence of TTX and 4-AP (Figure 1E). Together, these data indicated that all of the four main cell types in the superfical SC receive direct, monosynaptic input from V1, with wide-field neurons receiving the highest proportion of V1 input followed by stellate neurons, narrow-field neurons and horizontal neurons.

We next investigated the properties of the V1 input to the different cell types in the superficial SC. To examine this, we measured the properties of EPSPs evoked by optogenetic stimulation of same strength and duration in all four cell types (Figures 4A–D, right; duration: 2 ms, intensity: 2.9 mW). Optogenetic stimulation of V1 input to wide-field neurons evoked subthreshold EPSPs in the majority of neurons (65%, 35 out of 54), although a significant proportion of wide-field neurons responded to V1 input by generating action potentials, indicating powerful input from V1 (Figure 4E; 35%; 19 out of 54). In those wide-field neurons that responded to optogenetic stimulation of V1 input with subthreshold responses, EPSP amplitudes were distributed over a wide range, with the majority of EPSPs having a relatively low average amplitude (Figure 4E; 4.3 ± 0.6 mV). Action potentials were only evoked by optogenetic stimulation of V1 input in small percentage of narrow-field neurons (22%, 2 out of 9), with the majority of narrow-field neurons responding with subthreshold EPSPs (Figure 4E; 78%; 7 out of 9). In those narrow-field neurons that responded to optogenetic stimulation of V1 input with subthreshold responses, EPSP amplitudes were distributed over a slightly wider range, with a higher average amplitude (Figure 4E; 9.3 ± 3.0 mV). All responses to optogenetic stimulation of V1 input in horizontal neurons were subthreshold, again distributed over a wide range, with intermediate average amplitude (Figure 4E; 6.6 ± 2.0 mV). Finally, only one stellate neuron responded to optogenetic stimulation of V1 input by generating an action potential, with the majority of stellate neurons generating relatively small EPSPs (Figure 4E; 4.96 ± 1.47 mV). With regard to EPSP half-width (EPSP duration at half EPSP amplitude) and rise time (time taken to go from 10% to the 90% of EPSP amplitude) we found that wide-field neurons had a statistically significantly shorter EPSP half-width compared to horizontal and stellate neurons (Figure 4F; 31 ± 2.7 ms compared to 53 ± 11 ms and 66 ± 9.3 ms, respectively). No statistical difference in EPSP rise time was observed between the four cell types (Figure 4G). In summary, these data indicate that while there is a lot of variability in the strength of V1 input to all four cell types, wide-field neurons receive the strongest input, with the highest percentage of cells generating action potentials in response to V1 input. The properties of EPSPs in the difference cell types were similar, although EPSPs in wide-field neurons had the shortest duration.

## Discussion

**Figure 5:**
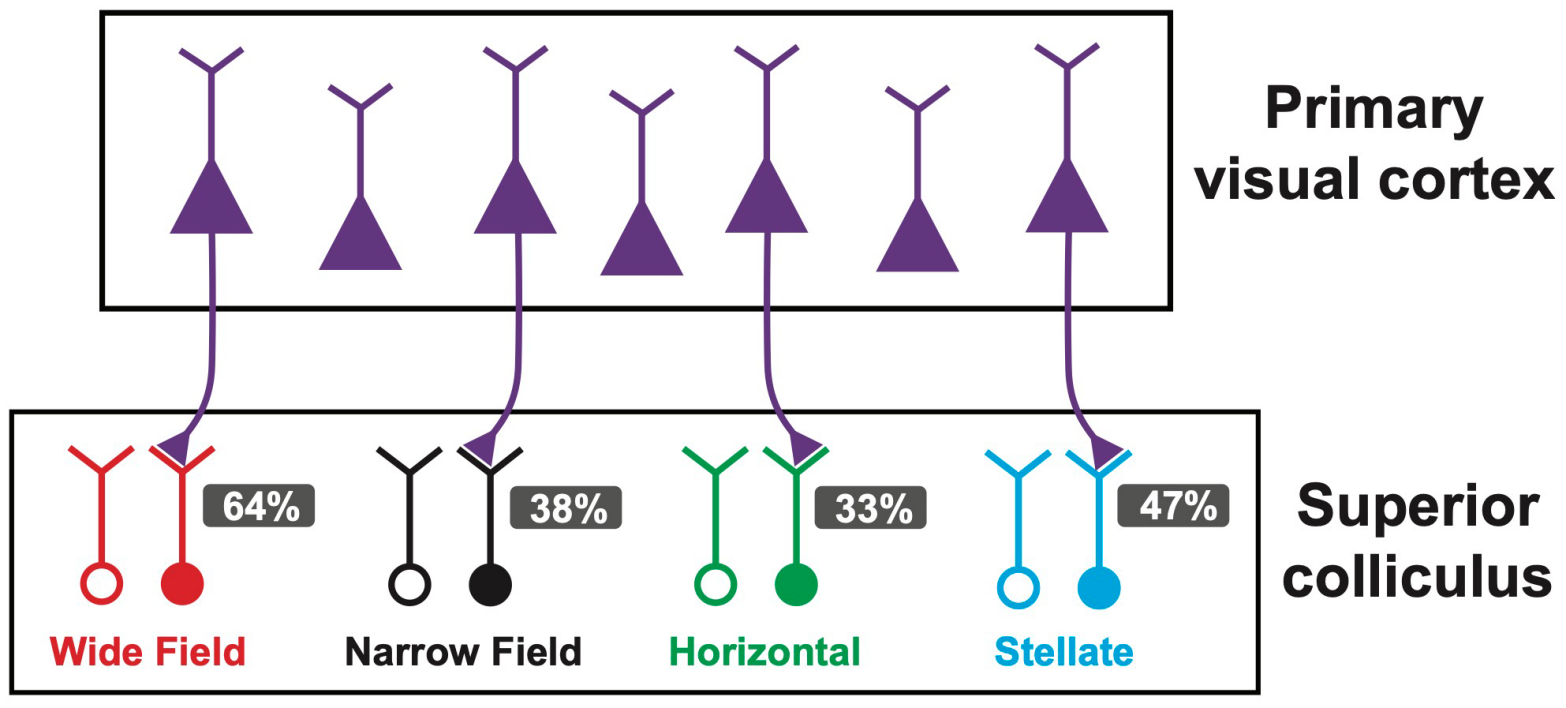
Summary schematic of primary visual cortex (V1) to superior colliculus (SC) connectivity for the four main cells types in superficial SC. V1 neurons project to all four cell types in SC. Different cell types are indicated by colour. The percentage of each cell type receiving V1 input is indicated. For each SC cell type, open circles represent somata of the population not receiving V1 input (“non-responders”), whereas solid circles represent somata of the population receiving V1 input (“responders”).

In this study, we investigated the connectivity between V1 and SC at the single cell level by combining optogenetic activation of V1 axons with whole-cell recording from different neuronal cell types in the superficial layers of the SC. We classified cell types into four main types based on electrophysiological and morphological properties – wide-field, narrow-field, horizontal and stellate. We found that V1 terminals made monosynaptic connections with all four cell types, with wide-field neurons receiving the highest fraction of V1 input followed by stellate neurons, narrow-field neurons and horizontal neurons. The majority of SC neurons showed subthreshold EPSPs in response to the optogenetic activation of V1 axons regardless of the cell type, with wide-field neurons the most likely to fire action potentials. These data suggest that V1 input to wide-field neurons in the SC is both more abundant and stronger than to other cells types. Our results are summarized in Figure 5.

### Cell types within the superficial layers of SC

Neurons in the superficial layers of SC have been classified into five groups previously based only on morphological properties (Langer and Lund, 1974; Mooney et al., 1985). However, a more recent study classified SC neurons into four groups due to a strong correspondence between their morphological and electrophysiological properties (Gale and Murphy, 2014, 2018). The fifth cell type – marginal neurons – are located most superficially, with dendrites ventrally extending toward the deeper SC (Langer and Lund, 1974). Earlier work suggests they have indistinguishable electrophysiological properties from stellate neurons (Gale and Murphy, 2014, 2018). Based on the earlier work by Gale and Murphy (2014, 2018), we classified SC neurons into four groups, although very recent work suggests this is an oversimplification (Li and Meister, 2023; Liu et al., 2023). Assuming four main cell types we found perfect (100%) alignment of our classification of wide-field and horizontal neurons based on their morphological and electrophysiological properties using unsupervised hierarchical cluster analysis. There was weaker correspondence, however, between the electrophysiological and morphological properties of narrow-field and stellate neurons. This finding is best explained by similarities in morphological and/or electrophysiological properties of these two cell types, leading to misidentification of either their morphology using the approach outlined in Figure 2 or allocation into incorrect clusters during the hierarchical cluster analysis in Figure 3.

Neurons classified as wide-field were characterized by the strongest depolarizing sag in response to hyperpolarizing current pulses (Figure 3D), consistent with earlier work showing strong expression of hyperpolarization-activated cyclic nucleotide-gated (HCN) channels in wide-field neurons (Endo et al., 2008). This earlier work suggests that HCN channels in wide-field neurons facilitate dendritic initiation and/or propagation of action potentials and thereby enhance action potential generation (Endo et al., 2008). This may contribute to the high percentage of wide-field neurons in our study generating action potentials in response to optogenetic stimulation of V1 inputs.

### Input circuitry from V1 to the superficial layers of SC

By classifying cells into different cell types based on their morphological and electrophysiological properties we show that all of the four main cell types in the superficial SC receive V1 input. In our experiments, on average 52% of neurons in the superficial SC received monosynaptic input from V1 (80 out of 153 neurons). This result is similar to a recent study reporting that on average 43% of neurons in the superficial SC receive V1 input (Masterson et al., 2019). Also consistent with the study by Masterson et al. (2019), we found that V1 was more likely to target wide-field neurons than other neuronal cell types. Masterson et al. (2019) found that V1 input to the SC targets both excitatory and inhibitory neurons in the SC, consistent with an earlier report (Zingg et al., 2017). Interestingly, Masterson et al. (2019) found that only 11% of inhibitory (GAD67-positive) GABAergic neurons in the superficial SC neurons received input from V1 input. Consistent with this result, we found that horizontal neurons, which previous work suggests are GABAergic (Gale and Murphy, 2014, 2018), receive the least V1 input of the four cell types in the superficial SC (Figure 4). Together, these results suggest that V1 primarily targets excitatory neurons, with the strongest projection to wide-field neurons.

The dendrites of wide-field neurons extend laterally as well as to the dorsal surface of the SC (see Figure 2A). This morphological feature allows wide-field neurons to receive visual input over a large part of the receptive field (Gale and Murphy, 2014) and may well explain why wide field neurons receive more V1 input than any other cell type in the superficial SC. As wide-field neurons also receive direct visual input from the retina (Tsai et al., 2022), one question for future research would be to determine to what extent V1 and retinal input to wide-field neurons is retinotopically mapped to the same dendrites of wide-field neurons.

EPSPs properties of V1 input to the superficial SC were similar across the different cell types (Figure 4), although EPSPs had briefer half-widths in wide-field neurons. Given earlier work showing EPSP rise time and half-width are correlated with on synaptic location (Rall, 1967; Letzkus et al., 2006), the finding that EPSP rise times are similar across the different cell types suggests that V1 input to the SC targets similar somatodendritic locations in these four cells types. With regard to the shorter half-width of EPSPs in wide-field neurons, this is likely due to the high level of expression of HCN channels in these neurons (Williams and Stuart, 2000; Endo et al., 2008).

Finally, the finding that a significant proportion of neurons in each cell type did not receive V1 input, despite the fact that activation of V1 input could generate action potentials in all four cell types except horizontal cells (which are thought to be inhibitory), suggests that the interconnectivity between excitatory neurons within the superficial SC is sparse. Alternatively, across the different cell types there may be separate, but interconnected, populations: One population which receives V1 input, and another that does not (Figure 5).

### Functional implications of cell-type based SC circuits

Previous work indicates that the SC plays a critical role in the generation of defensive behaviours in mice in response to threatening visual stimuli. When mice are presented with visual stimuli representing the rapid approach of a predator, or stimuli representing a predatory cruising overhead, mice exhibit escape or freezing behaviours, retrospectively, with the type of defensive behaviour dependent on the stimulus (Yilmaz and Meister, 2013; De Franceschi et al., 2016).

While there is evidence that the direct retinal projection to the SC is involved in the generation of escape behaviours to looming stimuli (Kim et al., 2020; Cai et al., 2021; Yuan et al., 2023), other work indicates that the V1 projection to the SC neurons modulates responses in the SC to looming stimuli (Zhao et al., 2014). The V1 projection to the SC is also thought to play a role in the generation of freezing behaviours (Liang et al., 2015; Zingg et al., 2017). Consistent with this idea, recent work indicates that V1-recipient SC neurons send projections to the lateral posterior thalamic nuclei (Zingg et al., 2017), which is known to be involved in the generation of freezing behaviours in mice (Wei et al., 2015), and optogenetic activation of the axon terminals of V1-recipient SC neurons in the LP triggers freezing (Zingg et al., 2017). Wide-field neurons are the only cell type in the superficial layers of the SC that project to the LP (Gale and Murphy, 2014, 2018). Furthermore, at a functional level wide-field neurons preferentially respond to small, slowing moving dots representing a predatory cruising overhead (Gale and Murphy, 2014), which drive freezing behaviours (De Franceschi et al., 2016). Together, these data suggest that the V1-SC-LP pathway is critical for the initiation of freezing in response to threatening visual stimuli (Zingg et al., 2017). Our findings, together with the earlier work by Masterson et al. (2019), provide further support for this idea by showing that wide-field neurons receive the strongest projection from V1. Finally, as LP projects to V1 (Juavinett et al., 2020), the V1-SC-LP pathway creates a positive feedback loop back to V1, which may act to reinforce or enhance freezing. In addition to this pathway, other work indicates there is an alternative pathway to visual cortex from SC via LP to the postrhinal cortex (Beltramo and Scanziani, 2019). This pathway is independent of V1 and particular sensitive to moving objects, so may also play a role in defensive behaviours to threatening visual stimuli.

Apart from wide-field neurons, what is the potential function of the V1 projection to other cell types in the superficial SC? A previous study showed that parvalbumin-positive (PV^+^) SC neurons in the superficial SC provide excitatory input to the parabigeminal nucleus (PBG), evoking escape behaviours in freely moving mice (Shang et al., 2015). PV^+^ neurons in the superficial layers of SC have been classified into three major groups based on their morphological properties: stellate, narrow-field and horizontal neurons (Villalobos et al., 2018). All three of these cell types are known to target the PBG (Gale and Murphy, 2014, 2018). As we show that all three of these cell types also receive V1 input, it is possible that the V1 projection to one or more of these cell types plays a role in inducing and/or modulating escaping behaviours. Consistent with this idea, as mentioned above, the V1 projection to the SC modulates responses in the SC to looming stimuli (Zhao et al., 2014), which are known to drive escape behaviours in mice (Yilmaz and Meister, 2013; De Franceschi et al., 2016). Against the idea that V1 input to the SC drives escape, optogenetic activation of the axons of V1-recipient SC neurons in the PBG does not induce escape behaviour (Zingg et al., 2017). This may suggest that the PV^+^ SC neurons that evoke escape behaviours represent the population of narrow-field, stellate or horizontal neurons that do not receive direct input from V1 (Figure 5). In conclusion, the results described above suggest that the V1 projection to SC plays an important role in driving freezing, whereas it may be more important for modulating rather than intiating escape.

In summary, we characterize the V1 input to specific cell types in the SC, finding that all four cell types in the superficial layers of the SC received monosynaptic (direct) input from V1. Wide-field neurons were more likely than other cell types to receive V1 input, with this input being the most powerful. Furthermore, our data suggest that within each cell type there may be different populations: Those that receive monosynaptic V1 input and those that do not. This data provides cell specificity of the V1 to SC projection, increasing our understanding of how visual information is processed in the superior colliculus at the single cell level.

## Data availability statement

The raw data supporting the conclusions of this article will be made available by the authors, without undue reservation.

## Ethics statement

The animal study was approved by Australian National University Animal Experimentation Ethics Committee. The study was conducted in accordance with the local legislation and institutional requirements.

## Author contributions

SJ: Formal analysis, Investigation, Writing – original draft, Writing – review & editing. SH: Formal analysis, Investigation, Methodology, Supervision, Writing – review & editing. GS: Conceptualization, Funding acquisition, Methodology, Project administration, Resources, Supervision, Writing – original draft, Writing – review & editing.
