# Erratum: Characterization of primary visual cortex input to specific cell types in the superior colliculus

**DOI:** 10.3389/fnana.2023.1346294

**Published:** 2023-12-13

**Authors:** Frontiers Production Office

**Affiliations:** Frontiers Media SA, Lausanne, Switzerland

**Keywords:** superior colliculus, visual cortex, vision, optogenetics, patch clamp

Due to a production error, the DOI in the header of each page of the PDF was incorrectly given as doi: 10.3389/fpubh.2023.1056191. The correct DOI is doi: 10.3389/fnana.2023.1282941.

The publisher apologizes for this mistake. The original article has been updated.

